# Improved Survival of a HER2-Positive Metastatic Breast Cancer Patient Following a Personalized Peptide Immunization

**DOI:** 10.3390/vaccines11061023

**Published:** 2023-05-25

**Authors:** Wolfgang Schönharting, Tim Roehnisch, Mehdi Manoochehri, Jan Christoph, Marie Sieger, Mauro Nogueira, Mari Carmen Martos-Contreras, Meik Kunz

**Affiliations:** 1 PMCR GmbH, 76135 Karlsruhe, Germany; 2 Interdisziplinäres Onkologisches Zentrum (IOZ), 80336 Munich, Germany; 3 AG Bio-Medical Data Science, Martin-Luther-Universität Halle-Wittenberg, 06108 Halle, Germany; 4 Chair of Medical Informatics, Friedrich-Alexander University (FAU) of Erlangen-Nürnberg, 91054 Erlangen, Germany

**Keywords:** breast cancer, case report, neoantigen, immunotherapy, bioinformatics, peptide

## Abstract

Cancer neoantigens that arise from somatic mutations have emerged as important targets for personalized immunization. Here, we report an improved overall survival of a HER2-positive metastatic breast cancer patient using a bioinformatic-based personalized peptide immunization called BITAP (BioInformatic Tumor Address Peptides). The epitopes were predicted using our in-house bioinformatic pipeline, and the immunogenicity was tested by IFN-γ ELISPOT and intracellular cytokine staining assays. In total, a significant peptide-specific T-cell response was detected against 18 out of the 76 (≈24%) tested peptides. The patient’s follow-up by measuring serologic markers showed a significant reduction in the tumor marker levels following BITAP immunization. Along with standard treatment, the patient treated with the BITAP showed stable disease with a remarkably improved overall survival, and no serious treatment-related adverse effects. In conclusion, our findings suggest that BITAP immunization is feasible, and safe, and may induce tumor regressions in patients with HER2-positive subsets of breast cancer.

## 1. Introduction

Breast cancer (BC) is the most common cancer type among women accounting for almost one-third of all diagnosed cancers. It also accounts for 15% of cancer-related death numbers in women, holding second place after lung cancer [1]. Depending on the clinical and molecular features of breast tumors, the patients are usually treated with chemotherapy, hormonal therapy, and/or radiotherapy; however, on average, one out of three patients will die of their disease [2]. Therefore, the development of new therapeutic approaches for breast cancer, either as a combination therapy or first-line therapy with lower side effects, is warranted. 

Immunotherapy, using Immune checkpoint inhibitors (ICI), has revolutionized the treatment of several cancer types over the past decade and prolonged the survival of cancer patients [3]. Despite the therapeutic effect of immune checkpoint blockade in several tumor entities with high mutation burdens, such as melanoma and non-small cell lung carcinoma, most of patients with solid tumors such as BC, did not achieve an objective response following treatment with anti-PD-1/PD-L1 monotherapy [4]. 

HER2-positive BC constitutes 15–20% of newly diagnosed invasive breast carcinomas [5]. HER2-blocking therapies in combination with chemotherapy represent the standard first-line treatment for HER2-positive metastatic BC [6]; however, the disease will eventually progress in most cases. Active immunotherapy by using generalized peptide cancer vaccines against HER2 shows no promising results [7]. Among BC subtypes, HER2-positive and triple-negative breast cancer (TNBC) are more immunogenic due to a higher mutational burden which suggests an expected benefit from immunotherapy in these two aggressive BC subtypes [8,9]. Neoantigens, which arise from somatic mutations in cancer cells, are valuable targets for individualized cancer immunization [3]. These tumor-specific antigens (TSA), along with tumor-associated antigens (TAA), comprise targets for designing personalized cancer immunotherapy [10]. Here, we report our findings on a patient with HER2-positive metastatic BC treated with a bioinformatics-derived personalized peptide vaccination. The vaccine was designed based on our in-house BITAP bioinformatic platform using the exome and transcriptome analysis of the tumor and normal tissues. Several cycles of injections with BITAP immunization peptide pools led to stable disease with a remarkable improvement in the patient’s overall survival. 

## 2. Materials and Methods

### 2.1. Case Presentation 

**Figure 1:**
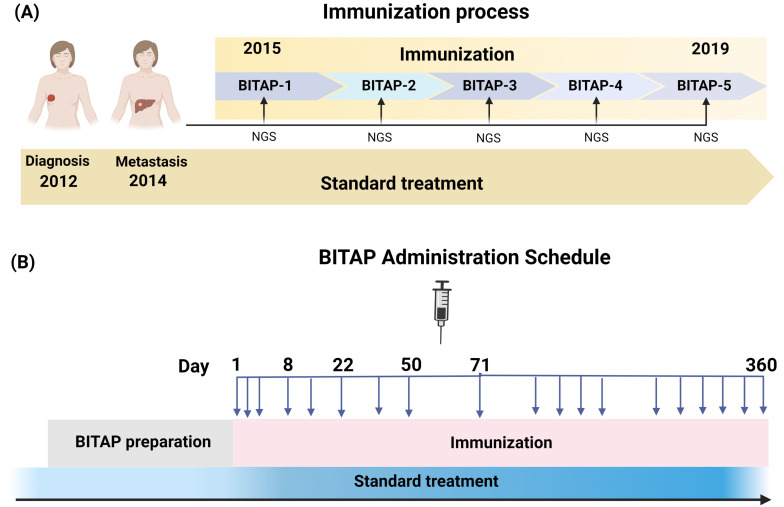
The immunization process of BC patient by interactive development of multi-peptide BITAP pools. (**A**) The patient received different BITAP peptide pools (BITAP-1–BITAP-5) containing different peptide pools over 5 years along with standard of care therapy. Each BITAP pool was developed by a new NGS analysis of tumor at different years. (**B**) The BITAP administration schedule. The patient generally received 19 subcutaneous injections of each BITAP peptide pool.

BrCa-02 patient. A 40-year-old female patient was diagnosed with metastatic carcinoma of the right breast in 2012. The pathological evaluation defined pT2, pN1sn pN15(3/5) G2, and M0 stage. Immunohistochemical analysis of the tumor showed receptor negative (ER−, PR−) and HER-2/neu positive with a Ki-67 of 55%. The patient’s mother had also been diagnosed with breast cancer at the age of 45 years old. Standard treatment was started by surgical removal of the sentinel node of the right axillary before primary systemic therapy. The primary systemic neoadjuvant chemotherapy was started with six cycles of carboplatin, docetaxel, and trastuzumab (6× TCH) in 2012. After six cycles of chemotherapy, adjuvant combined therapy with radiation and trastuzumab continued for a year. In 2014, the patient was again referred to the hospital and diagnosed with metastasis in the left lobes of the liver. Pathological and immunohistochemical analysis showed invasive ductal carcinoma with ER−, PR−, Her2/neu+, and Ki-67 of 60%. As first-line standard therapies were not successful, the patient underwent personalized peptide immunization using five BITAP immunization peptide pools over five years (Figure 1A,B). At the time of starting the vaccination and considering clinical and historical control data, the patient was expected to have an OS of probably 6 months.

### 2.2. Whole Exome Sequencing 

The whole exome sequencing was performed on DNA isolated from the tumor and blood samples. Library preparation was performed using Sure Select XT Library Prep Kit (Agilent Technologies, Santa Clara, CA, USA) and then sequenced on Illumina HiSeq 4000 to produce 100 bp paired-end reads. 

### 2.3. Transcriptome Profiling

Transcriptome profiling was performed using either RNA microarray or NGS. In summary, RNA was isolated from tumor tissue and quality controlled using the 2100 Bioanalyzer (Agilent) following the manufacturer’s protocol. Library was prepared using TruSeq Standard mRNA LT kit and sequenced on Illumina HiSeq 4000 to produce 100 bp paired-end reads. For the microarrays, RNA amplification, labeling, and hybridization were performed using a custom high-density 44 K oligo array (Agilent Technologies). Microarray data normalization and quality control were performed using GeneSpring GX 13.0 (Agilent Technologies, Santa Clara, CA, USA). The obtained expression values were submitted to Student’s unpaired *t*-test, and *p* values were adjusted using the Benjamini–Hochberg multiple testing correction. 

### 2.4. HLA Typing

The patient’s HLA alleles were assessed by Labcorp using PCR sequence-based typing. The patient-specific analysis resulted in the following assignment of human HLA alleles: HLA-A*02:01, A*24:02, B*08:01, B*51:01, C*07:01, C*15:02; HLA-DRB1*09:01; DRB1*16:01, DQB1*03:03, and DQB1*05:02. 

### 2.5. Bioinformatic Characterization of Neoantigens 

The selection of neoantigen-containing peptides in the immunization peptide pool was performed according to our in-house BITAP bioinformatics analysis pipeline and using new sequencing data of metastatic tumors (clinical course). Briefly, WES reads were aligned to UCSC human reference genome hg19 (GRCh37), and then duplicate reads were identified. The somatic mutations were detected and manually confirmed, and the expression level of genes corresponding to somatic mutations was defined using transcriptome data. For each selected mutation, all possible peptides containing mutated amino acids were extracted, and their binding affinity to the corresponding patient’s human leukocyte antigen (HLA) class I alleles (HLA-A, HLA-B, and HLA-C) and HLA class II alleles were predicted. Together with other criteria, the peptides with desired MHC binding affinities were considered for next steps to finalize the selection of peptide pools for immunization. 

### 2.6. Peptide Manufacturing and Injection 

The BITAP peptide pools contain various numbers of short and long synthetic peptides for the injections in every immunization cycle. The personalized peptides were synthesized by the standard solid-phase synthetic peptide chemistry and purified using reverse-phase high-performance liquid chromatography with >90% purity. The peptides were mixed in a 33% DMSO/H_2_O injection solution and divided into several vial units. The BITAP immunization peptide pool consisted of 300 µg per peptide emulsified in Montanide ISA 51 VG as an adjuvant, which then was applied subcutaneously (s.c.) at each date at minimum 2 locations (left/right upper arms and tights). Before vaccine injection, 250 mg imiquimod (Opdivo) was applied to the skin at the injection sites; and in case of high PD-L1 expression, 1300 μg nivolumab was applied subcutaneously next to the injection site 30 min before injection. In total and along with standard treatment, five cycles of BITAP immunization (BITAP-1 to BITAP-5) with various numbers of peptides were applied over five years from 2015 to 2019. In general, 19 injections were applied on days 1, 2, 3, 8, 15, 22, 36, 50, and 71; and ten additional immunizations were applied every 3 weeks until day 365. 

### 2.7. Immunogenicity Testing and Patient Follow-Up

The T-cell responses to the peptides were monitored in peripheral blood mononuclear cells (PBMCs) isolated from blood drawn before vaccination. Accordingly, 60 mL of blood was taken from the patient, and PBMCs for immunogenicity testing were isolated by Ficoll density gradient centrifugation. In vitro stimulation was performed using 5 million cells in 4 individual wells until day 12 using 120 U/mL interleukin (IL)-2. After 12 days of stimulation, responses to peptides were monitored by IFN-γ ELISPOT and, in some cases, were confirmed by Intracellular Cytokine Staining (ICS). The immunogenicity of the peptide therapy was determined by assessing the T-cell response by applying amplified IFN-γ ELISpot according to CIMT Immunoguiding Program (CIP) guidelines. All tests were performed in duplicate or triplicate and included negative (10% DMSO) and positive controls (10 μg/mL PHA-L). The spots were counted using the ImmunoSpot Series 2.0 Analyzer (CTL, Cleveland, OH, USA), and ELISPOT responses were considered positive when the numbers of IFN-γ–secreting cells were at least 2-fold above the negative control (medium) and with a minimum of 50 detected spots. The patient underwent regular follow-up evaluations by liver (γ-GT, Alkaline phosphatase, Bilirubin, GOT, and GPT) and tumor (CA 15-03 and CEA) serologic markers. 

## 3. Results

### 3.1. Identification of Tumor Antigens and BITAP Preparation

The BITAP immunization peptide pools were prepared according to our in-house development workflow. To obtain the tumor-specific antigens (TSA) or neoantigens, the DNA from tumor tissue and the PBMCs were subjected to whole exome sequencing and processed by our in-house bioinformatic pipeline. The mutations which are expressed at the RNA level, having high binding affinity prediction to the respected HLA class I or class II alleles, and occurring in functionally important cancer-associated genes/pathways were prioritized for selection. The mRNA expression data of tumor tissue samples were quantified using RNA microarrays and RNA sequencing. To identify TAAs, the cancer hallmark genes with known function for tumor proliferation, angiogenesis, and metastasis that do not or only slightly express in other tissues, and cancer–testis antigens, which are not expressed in healthy adult tissues but have high expression in the tumor, were prioritized. In total, 76 epitopes, including TSAs and TAAs, were selected for synthesizing antigen peptides within the five BITAP immunization peptide pools (Supplementary Table S1). 

### 3.2. In Vitro Tests Shows Strong Immunogenicity of Several Selected Peptides 

**Figure 2:**
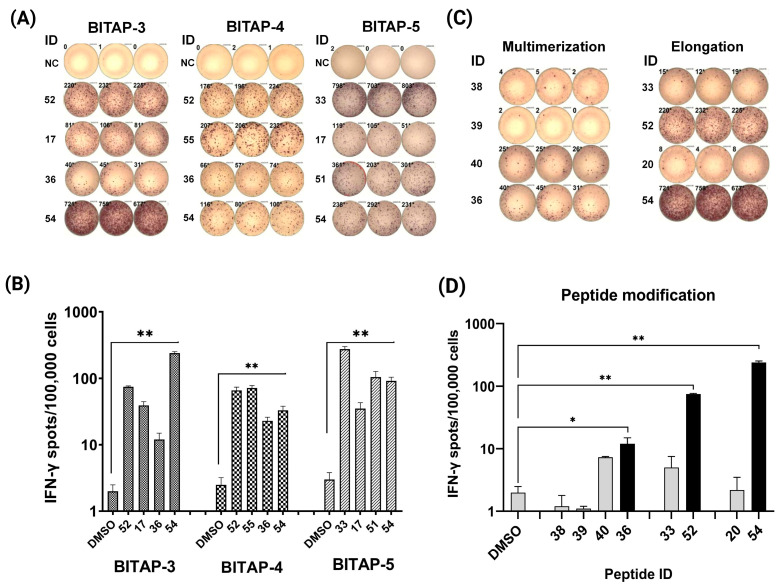
Immunogenicity testing of the predicted peptides. (**A**) IFN-γ ELISPOT showed changes in peptide-specific IFN-γ secretion by PBMCs in response to different peptides and BITAP peptide pools following 12-day stimulation with the peptides. (**B**) Histogram of IFN-γ ELISPOT assay for selected peptides. (**C**) IFN-γ ELISPOT of modified (IDs: 36, 52, and 54) and short (IDs: 38, 39, 40, 33, and 20) peptides. (**D**) Histogram of IFN-γ ELISPOT assay for modified and short peptides. * *p*  ≤ 0.05; ** *p* ≤  0.01.

The immunogenicity of all selected peptides (Supplementary Table S1) was tested by T-cell responses in peripheral blood mononuclear cells (PBMCs) by IFN-γ ELISPOT and intracellular cytokine staining. In total, 76 peptides, including 66 class I and 10 class II peptides, were administered in five cycles of BITAP immunizations. The ELISPOT results showed that ≈17% of class I (n = 11) peptides and 70% of class II peptides (n = 7) significantly increased the IFN-γ production in T-cells compared with that of negative controls. As a representative, four immunogenic peptides with positive ELISPOT results in three BITAP immunizations are shown (Figure 2A). The selected peptides showed a significant increase in T-cell activation (Figure 2B). Additionally, it was also showed that elongation of short peptide epitopes, either by including more amino acids from each side of the peptide or by epitope multimerization, significantly increased the immunogenicity of the epitopes (up to 14 times higher in ELISPOT assay) (Figure 2C,D). 

### 3.3. Peripheral Biomarker Monitoring Showed Therapy Response

**Figure 3:**
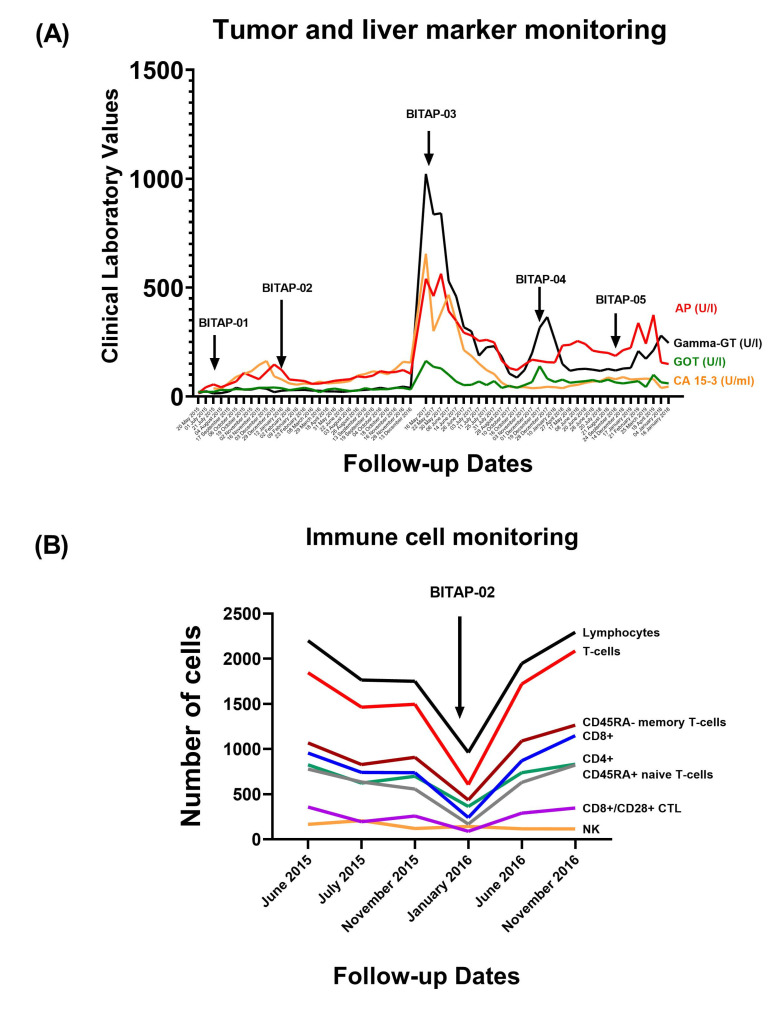
Monitoring of the patient during immunization. (**A**) The dynamic change in liver and tumor marker levels. (**B**) Immune cell monitoring in patient during BITAP immunization.

In order to determine the patient’s response to therapy, circulating biomarkers, including tumor marker (CA 15-3), liver markers (AP, Gamma-GT, and GOT), and blood immune cell counts, were investigated in the patient (Figure 3A,B). Tumor marker (CA 15-3) measurement showed a stable level during the immunization process, while it significantly increased after stopping the BITAP application in early 2017 (Figure 3A). Accordingly, re-starting the application of peptide pools in combination with standard treatment significantly decreased or stabilized the tumor/liver marker levels. In addition, immune cell monitoring showed an increase in the total number of lymphocytes (CD4+ and CD8+) following BITAP immunization (Figure 3B). 

## 4. Discussion

Here, we report one breast cancer case that underwent and benefited from individualized peptide-based immunotherapy using our in-house bioinformatics BITAP platform. The patient was diagnosed with metastatic breast cancer and suffered tumor progression following standard treatments. Based on the patient’s own mutanome and transcriptome by several sequencing times, the pools of tumor antigens were identified, and corresponding peptide combinations were calculated. The peptides within each BITAP were selected based on our in-house epitope prioritization pipeline and evaluated in vitro using T-cell assays. Along with standard treatment, the patient received a total of five treatment cycles of BITAP-based immunotherapy over several years, from 2015 to 2019. Fortunately, the patient benefited from BITAP peptide pools, and immunization cycles were safe and generally well tolerated, with mild to moderate local site reactions being the most frequent side effects. Since the BITAP was administered in combination with standard therapy, it is not possible to estimate the attribution of clinical response connected only to the BITAP peptide pools. However, historical control and clinical expectation data showed that the OS associated with the BITAP combined treatment was 60 months, which significantly improved relative to the standard clinical expectation. The regular patient follow-up using liver and tumor marker measurements was performed by blood sampling at various time points, which also showed significant decreases or stabilized peripheral biomarkers, together with an increase in the number of T-cells following BITAP application. An increase in T-cells, including CD4+ and CD8+ T-cells, as well as CTLs, after immunization indicates that the vaccine is successfully activating the immune system to respond to the antigenic peptide pool.

The immunogenicity of each of the peptide epitopes administered in this case was analyzed by IFN-γ release ELISPOT assay showing that ≈17% of class I peptides and 70% of class II peptides significantly increased the IFN-γ production in T-cells. In addition, the modifications of predicted short peptides, either by elongation or multimerization, significantly improved the immunogenicity of epitopes compared to single short peptides (up to 14 times greater immunogenic response in ELISPOT). This result is in agreement with previous findings highlighting the potential of synthetic long peptides as a more immunogenic vaccine platform in comparison with the exact short peptides of 8–10 amino acids in length [11]. 

In the current case report on a HER2-positive patient, Montanide ISA 51 VG was applied, which is the main adjuvant used in cancer vaccination trials [12]. The adverse effects of peptide vaccination consisted of pain and skin reactions, such as redness and swelling at the inject ion sites, which were tolerable. This suggests that peptide vaccination with the adjuvant might be applicable as a treatment for HER2-positive metastatic BC patients. In this BC patient, a personalized peptide-based immunotherapy showed a synergistic effect with the conventional treatment. Therefore, a combination therapy using chemotherapy/targeted therapy and active immunotherapy using neo-antigens might be beneficial for improving the survival of HER2-positive BC patients. The utility of neoantigen immunization in combination with immune checkpoint and chemotherapy was also tested as first-line treatment in a recent study on lung cancer and suggests a robust effect of peptide vaccination in combination with chemotherapy and anti-PD-1 [13].

Compared to other approaches, such as chemotherapy, targeted therapy, and ICI immunotherapy, cancer vaccines have several unique features. Cancer vaccines aim to stimulate the immune system to recognize and attack cancer cells while minimizing damage to healthy cells. They can also potentially provide long-term protection against cancer recurrence. In the context of HER2-positive breast cancer, several cancer vaccine approaches have been investigated, including peptide-based vaccines, dendritic cell-based vaccines, and RNA/DNA-based vaccines. While some of these approaches have shown promising results in preclinical studies and early-phase clinical trials, more research is needed to determine their efficacy and safety in larger, randomized trials [14].

## 5. Conclusions

In conclusion, peptide-based immunotherapy in combination with conventional targeted therapy and chemotherapy could be beneficial for improving the survival of patients with metastatic HER2-positive BC; however, future clinical trials are warranted to evaluate the effectiveness of BITAP immunotherapy on HER2-positive BC.

## Data Availability

The data are not publicly available due to privacy/ethical restrictions.

## Supplementary Materials

The following supporting information can be downloaded at https://www.mdpi.com/article/10.3390/vaccines11061023/s1. Table S1: The list of peptides, including TSAs and TAAs, which were included and tested within the five BITAP immunization cocktails, their respective corresponding gene name, and their response in ELISPOT experiments.
