# Supplementary material for: Improved Survival of a HER2-Positive Metastatic Breast Cancer Patient Following a Personalized Peptide Immunization
Source: Vaccines (Basel). 2023 May 25;11(6):1023. doi: 10.3390/vaccines11061023 (PMC10301538; doi:10.3390/vaccines11061023)
Supplement: Supplementary file 1 [file vaccines-11-01023-s001.zip › vaccines-2295637-supplementary.pdf]

vaccines

MDPI

Table S1. The list of peptides including TSAs and TAAs which were included and tested within the five *BITAP* immunization cocktails, their respective corresponding gene name and response in ELISPOT experiments.

Peptide ID	Sequence	Gene	HLA Class	Immunogenic (ELISPOT)				
				BITAP-1	BITAP-2	BITAP-3	BITAP-4	BITAP-5
01	GLDEAIRKV	GLYL2	I	No	-	-	-	No
02	RYSDSVGRVSF	CAN13	I	No	-	-	-	-
03	QIWDKILSV	DHRS2	I	No	-	-	-	-
04	GLISELKLA	SEHL2	I	No	-	-	-	-
05	IPAPPLSKV	NGAL	I	No	-	-	-	-
06	KSMDIVLTV	MUC5B	I	No	-	-	-	-
07	DAKLEPTNV	CDC6	I	No	-	-	-	-
08	MPNQAQMRI	ERBB2	I	No	-	-	-	-
09	**TYLPTNASLSF**	ERBB2	I	Yes	-	No	-	No
10	KIADFGWSV	AURKA	I	No	-	-	-	-
11	TTYIFRLAAKNRAG	PTPRF	II	No	-	-	-	-
12	**NEKVIEHIM**	S10A9	I	Yes	-	-	-	-
13	**KYSLIKGNF**	S10A8	I	Yes	-	-	-	-
14	TLFDYEVRL	UHRF1	I	No	-	-	-	-
15	**SYQKVIELF**	TOPK	I	Yes	-	-	-	No
16	PFLQASPHF	FA83A	I	No	-	-	-	-
17	**EDKKIDFSEFLSLLGDI**	S10A7	II	Yes	Yes	Yes	Yes	Yes
18	MEESHLNSNPYFWPSI	ZN384	II	No	-	-	-	-
19	SKMENPGSV	CAMK1	I	-	No	-	-	-
20	**SHKSPQLSV**	CD33	I	-	Yes	-	-	-
21	AAVAHMIEA	DENND4	I	-	No	-	-	-
22	CAHNASGYF	DIP2C	I	-	No	-	-	-
23	EETSNLSAL	HIVEP2	I	-	No	-	-	-
24	SEVLFIATA	LONP2	I	-	No	-	-	-
25	GAVNGPLVV	NASP	I	-	No	-	-	-
26	NALQLGLMV	PIGS	I	-	No	-	-	-
27	LQLGLMVPV	PIGS(vs)	I	-	No	-	-	-
28	YKSDLVQIY	PRKAG1	I	-	No	-	-	-
29	RHHSKRSQR	RBBP6	I	-	No	-	-	-
30	SAARASIEL	RCAN2	I	-	No	-	-	-
31	KCVNEDLKI	SAMD9L	I	-	No	-	-	-
32	QRSSHYHE	SND1	I	-	No	-	-	-
33	**DEDEIKWWW**	TP53BP2	I	-	Yes	No	Yes	Yes
34	NLEGVETQL	TRAP1	I	-	No	-	-	-
35	KTISLPPPI	AFF2	I	-	No	-	-	-

36	**KYIQESQALAKRSCGLFQ KLGEYYLQNAFL**	AFP	II	-	-	Yes	Yes	-
37	AYTKKAPQL	AFP	I	-	-	No	-	-
38	KYIQESQAL	AFP	I	-	-	No	-	-
39	RSCGLFQKL	AFP	I	-	-	No	-	-
40	EYYLQNAFL	AFP	I	-	-	No	-	-
41	LATIFFAQFV	AFP	I	-	-	No	-	-
42	PLFQVPEPV	AFP	I	-	-	No	-	-
43	KVNFTEIQKL	AFP	I	-	-	No	-	No
44	ILYPGGNKY	DKKL1	I	-	-	No	-	-
45	ALGAAGATRV	DKKL1	I	-	-	No	-	-
46	RLLILILSII	MAGEC1	I	-	-	No	-	-
47	KVWVQEHYL	MAGEC1	I	-	-	No	-	No
48	ILAVDGVLSV	SLC30A8	I	-	-	No	-	-
49	HETYGHKTPY	CCL28	I	-	-	No	-	No
50	LEQEIATY	KRT222	I	-	-	No	Yes	-
51	**VDLIVEYEAFPKPE**	KIT	II	-	-	No	Yes	Yes
52	**IHREDEDEIKWWWARLN**	TP53BP2	II	-	-	Yes	Yes	-
54	**STKYSHKSPQLSVHVTD**	CD33	II	-	-	Yes	Yes	Yes
55	**ATYSGAGYYLDLSIT**	PKD2	II	-	-	-	Yes	-
56	**HGSSFFLLILKRDSAFI**	ENTHD2	II	-	-	-	Yes	-
57	TASQRFIELGADIKRVK	ELL3	II	-	-	-	No	-
58	**DAVIVKLEI**	PKD2	I	-	-	-	Yes	-
59	**YYLDLSTTR**	PKD2	I	-	-	-	Yes	-
60	**ILFGISLREV**	MAGEC1	I	-	-	-	Yes	-
61	KLLLAEKAV	PLEC	I	-	-	-	No	-
62	RTPLSALCV	ASIP	I	-	-	-	No	No
63	LLGGNALKF	SALL3	I	-	-	-	No	-
64	WVHCAHNAS	DIP2C	I	-	-	-	No	-
65	MPLDDPLGR	ZC3H12C	I	-	-	-	No	-
66	RFIELGADI	ELL3	I	-	-	-	No	-
67	**KVVEFLAML**	MAGEC1	I	-	-	-	Yes	-
68	NLMEQPIKV	JUP	I	-	-	-	No	--
69	**FVNDKFMPL**	ZC3H12 A	I	-	-	-	Yes	-
70	CNCTRSLLR	MAP3K9	I	-	-	-	No	-
71	IYLEKLKTI	PLEC	I	-	-	-	No	-
72	WTLHNLSDV	JUP	I	-	-	-	No	-
73	**FLLILKRDS**	ENTHD2	I	-	-	-	Yes	-
74	GEIWVHCAH	DIP2C	I	-	-	-	No	-
75	LSTWDQPKK	TRANK1	I	-	-	-	No	-

76	VAKILTFPQ	POLR3A	I	-	-	-	No	-
